# Project Last Mile and the development of the Girl Champ brand in eSwatini: engaging the private sector to promote uptake of health services among adolescent girls and young women

**DOI:** 10.1080/17290376.2021.1894224

**Published:** 2021-03-08

**Authors:** Marie A. Brault, Sarah Christie, Sasha Aquino, Abigail Rendin, Amanda Manchia, Leslie A. Curry, Erika L. Linnander

**Affiliations:** a Department of Social and Behavioral Sciences, Yale School of Public Health, New Haven, CT, USA; b Department of Health Policy and Management, Yale School of Public Health, New Haven, CT, USA; c Global Health Leadership Initiative, Yale School of Public Health, New Haven, CT, USA; d School of Public Health, University of the Western Cape, Bellville, South Africa; e Olson Zaltman Associates, Pittsburgh, PA, USA; f Project Last Mile, Johannesburg, South Africa

**Keywords:** Adolescent girls and young women, public-private partnerships, sexual and reproductive health, qualitative research, eSwatini

## Abstract

In eSwatini and across sub-Saharan Africa, adolescent girls and young women (AGYW) are at significantly higher risk of HIV infection and poorer sexual and reproductive health (SRH) than their male counterparts. AGYW demonstrate low demand for SRH services, further contributing to poor outcomes. Strategic marketing approaches, including those used by multinational corporations, have potential to support demand creation for SRH services among AGYW, but there is limited empirical evidence on the direct application of private-sector strategic marketing approaches in this context. Therefore, we examined how Project Last Mile worked with eSwatini's Ministry of Heath to translate strategic marketing approaches from the Coca-Cola system to attract AGYW to SRH services. We present qualitative market research using the ZMET® methodology with 12 young Swazi women (ages 15–24), which informed development of a highly branded communication strategy consistent with other successful gain-framing approaches. Qualitative in-depth interviews with 19 stakeholders revealed receptivity to the market research findings, and highlighted local ownership over the strategic marketing process and brand. These results can inform similar efforts to translate strategic marketing to support demand generation in pursuit of public health goals to reduce HIV risk and improve SRH.

## Introduction

Adolescent girls and young women (AGYW) in Sub-Saharan Africa are at higher risk of HIV infection and poorer sexual and reproductive health (SRH) than their male counterparts (Cluver, Orkin, Meinck, Boyes, & Sherr, 2016; Mpondo, Ruiter, Schaafsma, van den Borne, & Reddy, 2018; Price et al., 2018; Schaefer et al., 2017; Sia et al., 2016). Despite higher sexual risk, HIV/STI testing and treatment rates remain low among youth (Asaolu et al., 2016; Chikwari, Dringus, & Ferrand, 2018; Sam-Agudu, Folayan, & Ezeanolue, 2016). Efforts to expand services and improve SRH among AGYW have been hindered by poor outreach and communication and, correspondingly, limited service demand and uptake (Amico & Bekker, 2019; Denno, Hoopes, & Chandra-Mouli, 2015; Lawrence, Struthers, & Van Hove, 2016; Mokomane, Mokhele, Mathews, & Makoae, 2017; Narasimhan, Pedersen, Ogilvie, & Vermund, 2017; Pretorius, Gibbs, Crankshaw, & Willan, 2015). Young women routinely cite demand-side issues as barriers to care, including limited awareness of available services, stigma associated with SRH, and negative experiences with healthcare providers (Jonas et al., 2018; Mokomane et al., 2017; Motuma, Syre, Egata, & Kenay, 2016; Muller, Rohrs, Hoffman-Wanderer, & Moult, 2016; Schriver, Meagley, Norris, Geary, & Stein, 2014).

Although demand creation through youth and community engagement has strong potential to improve uptake of youth-friendly services (Braeken & Rondinelli, 2012; Chandra-Mouli et al., 2017; Motuma et al., 2016; Mpondo et al., 2018), recent work has highlighted a paucity of examples that apply user-centered strategic marketing principles to meet this need in Sub-Saharan Africa (Schwartz et al., 2018). Furthermore, the potential to leverage private sector experience and expertise in strategic marketing to generate demand for services remains largely untested (Firestone, Rowe, Modi, & Sievers, 2017). Consequently, little is known about how diverse stakeholders may be meaningfully engaged to adapt private sector strategic marketing approaches for use in the public sector.

Therefore, we sought to document the efforts of Project Last Mile (PLM) in eSwatini, which leveraged strategic marketing skills from the Coca-Cola system to co-create and implement a communications strategy to promote uptake of HIV prevention and SRH services among AGYW, who are disproportionately impacted by HIV. We describe the process of co-developing the marketing strategy, including the results of the market research and stakeholder perceptions of the process, with careful attention to local receptivity, ownership, and translation of market research findings into a strategic marketing campaign to improve AGYW's demand for SRH services. Findings may be useful for others seeking to adopt private-sector marketing approaches to improve AGYW's engagement with health services in other low- and middle-income settings.

## Methods

This paper presents findings from two complementary qualitative methodologies: application of the Zaltman Metaphor Elicitation Technique (ZMET®) for client-centered market research with AGYW as an input into the strategic marketing approach, and in-depth key informant interviews to characterise stakeholder experiences with the translation of private-sector strategic marketing approaches into the public health sector. We present the study setting and background for the project, followed by methods for both components.

### Study setting and background

The Kingdom of eSwatini is a small country within the borders of South Africa, home to roughly 1.3 million people. Over a third of the population is between the ages of 10–24 years old ((UNFPA), 2019), and HIV prevalence is over five-times higher among young women 20–24 years old (20.9%) than young men (4.2%) (Ministry of Health Eswatini, 2019). The Ministry of Health (MoH) provides oversight of clinical services, including primary and preventative health services. Preventative health services include the Swaziland National AIDS Program (SNAP) and the Sexual Reproductive Health (SRH) program which spearheads the youth-friendly service initiative. The MoH also supports the Health Promotion Unit (HPU), responsible for national health communications. The National Emergency Response Council on HIV and AIDS (NERCHA) is a clearinghouse for HIV information and support, and facilitates coordination of national HIV prevention activities as a principal recipient of the Global Fund. This project was situated within the HPU of the MoH, supported by the Global Fund in partnership with NERCHA, and piloted in the Manzini region.

PLM is a public-private partnership with The Coca-Cola Company that shares best practices in supply chain management and strategic marketing to improve availability of medicines and uptake of health services in the public sector. Funded by a Global Development Alliance, the partnership engages directly with Ministries of Health throughout Africa on projects aligned with country priorities. In eSwatini, PLM applies the strategic marketing process adopted by large Consumer Packaged Goods (CPG) companies like Coca-Cola to tailor brand-building approaches to health communication and promotion. PLM works to build capacity within the MoH and its partners in a demand creation approach that is strategic, process-driven, and targeted. PLM also engaged Coca-Cola Marketing Africa for advice and connection to two suppliers: Olson Zaltman as the market research agency and FCB Africa for creative design.

### Stakeholder interview methods

As part of a global evaluation to explore the strengths, challenges, and lessons learned from the PLM partnership across multiple country settings (Linnander, LaMonaca, Brault, Vyavahare, & Curry, 2018), we conducted in-depth, in-person interviews with ‘key informants’ (those with deep experience with PLM in eSwatini) (Patton, 2002). Interviews followed a discussion guide with content probes to explore: participant receptivity, ownership, and translation of market research findings into a strategic marketing campaign to improve AGYW's demand for SRH services. Interviews lasted 45–60 min. All interviews were audio-taped and professionally transcribed by an independent transcription service.

### Stakeholder sampling

We used a purposeful sampling approach with snowball sampling to identify key informants (Patton, 2002); nineteen individuals, representing a variety of organisations (Table 1), participated in the interviews.

**Table 1.** Organisations represented in PLM evaluation interviews.

Organisation	Number of stakeholders
Public sector: Ministry of Health; Health Promotion Unit (HPU); Swaziland National AIDS Program (SNAP) and Adolescent Sexual Reproductive Health (ASRH) program	9
Private sector: Coca-Cola Marketing, FCB Africa, Olson Zaltman	6
PLM project team	3
Donor Organisation	1
Total	19

### Stakeholder data analysis

Two members of the research team (MB and SC) coded the data independently using Atlas.TI v8 and the constant comparison approach to identify themes inductively, overseen by a senior qualitative researcher (LC) (Glaser & Strauss, 1967). The codebook (see supplemental file) was iteratively developed over five drafts throughout the coding process. Team members were encouraged to challenge discrepant views, and the codebook included codes for disconfirming information. Thematic analyses were conducted through in-depth review of the code reports with reflection on the factors that facilitated and/or hindered support for the strategic marketing process, including local receptivity, relevance, and fit.

### Market research methods

**Figure 1.**
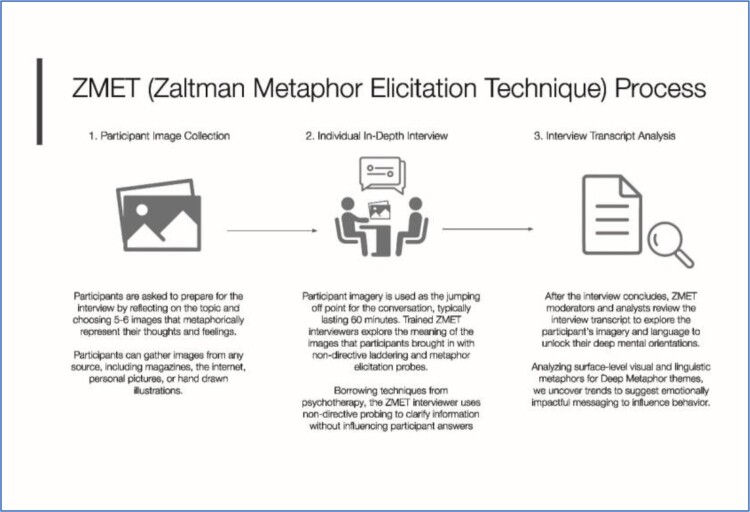
Overview of ZMET process.

Market research was conducted using the ZMET® methodology, a multi-disciplinary approach that uses metaphors and other nonliteral expressions to uncover people's unconscious emotional needs to develop targeted marketing and communications strategies (Zaltman, 1997, 2003; Zaltman & Coulter, 1995; Zaltman & Zaltman, 2008). The ZMET® methodology has also been used more recently in research exploring behaviour change and barriers/facilitators to use of academic and health services (Hancock & Foster, 2019; Khoo-Lattimore & Prideaux, 2013; Tseng & Chiu, 2020). For more details on the ZMET methodology, see the supplemental file. The current study used the ZMET approach to inform demand creation strategy development by uncovering the unconscious metaphors and socially shared associations (depicted in a consensus map) regarding HIV prevention among young women aged 15–24 years old in eSwatini. Figure 1 provides an overview of the ZMET process.

ZMET interviews were one-on-one in-depth discussions that lasted 90–120 min and consisted of four steps: Storytelling, Missed Images, Sensory Metaphors and a Vignette. Prior to the interview, participants were given the following assignment: ‘Please collect 5–7 images that express your thoughts and feelings about preventing HIV.’ These images, which participants could gather from any source or illustrate themselves, provided a starting point in the Storytelling step, where trained interviewers use non-directive probing techniques (e.g. laddering, metaphor elicitation, exploration (Reynolds & Gutman, 1988; Zaltman, 1997; Zaltman & Coulter, 1995), and storytelling (Boyd, 2009; László, 2008). In the Missing Images step, participants were asked if there were any concepts for which they could not find a physical image. If so, this was followed by similar probes outlined in the Storytelling Step. For Sensory Metaphors, participants were asked for sensorial metaphors (e.g. taste, touch, smell) that represented HIV prevention. Finally, in the Vignette, participants were asked to create a story that included three characters: Themselves, Their Health, and HIV Prevention to understand the personifications of each character, and the relationships between each character. Interviews were conducted in the participant's preferred language, and translated and transcribed verbatim in English for analysis.

### ZMET sampling

We recruited 12 participants from within and around the city of Manzini using a random route recruiting approach for one-on-one ZMET interviews (Table 2). Inclusion criteria were: between 15 and 24 years of age, living in eSwatini, and self-reported either negative or unsure of their HIV status. The selection of participants allowed for a natural mix of young women who had been sexually active or inactive within the last six months for the younger cohort (15–19 years old), with all of the participants being sexually active within the last six months for the older cohort (20–24 years old). The sample size was determined based on previous empirical market research findings that, for in-depth one-on-one interviews, 8 participants per consumer segment is adequate for saturation (Griffin & Hauser, 1993). As it was not necessary to identify further differences in segmentation (i.e. older or younger adolescents) or regionality, the sample size of 12 was appropriate.

**Table 2.** Participants in ZMET research, by age and origin.

Origin, age	Number of female respondents
Rural, 15–19 years old	2
Urban, 15–19 years old	4
Rural, 20–24 years old	4
Urban, 20–24 years old	2
Total	12

### ZMET data analysis

The ZMET analysis process uncovers the thought patterns from respondents’ images and interview responses, revealing deeper emotional meanings and unconscious mental orientations about how people frame the topic. For this study, the ZMET analysis process included several analyses from distinct steps in the interview process to help uncover convergent validity in the data (Damasio, 2018; McGilchrist, 2019; Zaltman, 2003, 2016). Three trained researchers independently coded the Storytelling and Missing Images sections of the transcripts using a proprietary software to produce a consensus map that outlines how contexts, behaviours, and their consequences (functional, social, psychological and emotional outcomes) are interrelated in the minds of participants. For the trend and themes analysis, researchers coded the Storytelling and Missing Images sections to identify metaphors, trends and themes. Researchers analysed the sensory metaphors, personifications and stories derived from the Sensory and Vignette steps along with images participants brought to the interview to identify metaphors, trends and themes.

### Ethics and consent

The ZMET market research protocol was approved by the MoH research ethics board. For minors (<18 years of age), written parental consent and young women's verbal assent to participate were obtained. For participants 18 years and older, verbal informed consent was obtained. Participants were informed that data they provided may be published, but any published information would be deidentified. The stakeholder study protocol was approved by the Yale University Human Research Protection Program (HRPP) Human Subjects Committee and endorsed by the MoH. All key informants provided verbal informed consent to participate.

## Results

The results from the two study components – market research and stakeholder interviews – are integrated to illustrate the process of developing the communication strategy (Table 3), with the stakeholder interview data bookending the ZMET market research data. The results are presented chronologically, mirroring the development of the communication strategy: (1) PLM's initial engagement in eSwatini, (2) collection and analysis of market research data using the ZMET approach to identify metaphors and perceptions of HIV prevention, (3) presentation of market research findings and receptivity, and (4) the resulting Girl Champ strategy.

**Table 3.** Key themes from the stakeholder interviews and market research.

Main theme	Subthemes
PLM achieved ownership and buy-in across diverse partners	• Proactive engagement with local stakeholders was achieved through establishment of a technical working group
	• Stakeholders’ commitment to building capacity within the HPU seen as unique and beneficial
	• Application of proven approaches from the private sector resonated with partners.
ZMET uncovered AGYW perspectives on power dynamics, aspirations, and identity related to HIV prevention	• The metaphor of a **tree of prevention** emerged, in which strength was derived from strong roots – environmental and social factors that supported an HIV prevention mindset. • AGYW compared HIV prevention knowledge to **freedom and light**, which they needed to remain healthy • The metaphor of **connection** emerged as young women expressed desire for supportive and empathetic female adults, including healthcare workers, and described judgement and stigmatisation they feared in seeking information and treatment for sexual health issues. • Imagery of **armoured plants and fierce animals** reflected AGYW desire for control over their future and strength to withstand societal pressures
Stakeholders Described Strong Receptivity to Market Research Findings	• Robust insights provided valuable nuance and context • Findings were readily translated into a communications strategy

### PLM achieved ownership and buy-in across diverse partners

Key informants across sectors valued PLM's emphasis on identifying local champions and encouraging the eSwatini MoH to ‘own the work’ through creation of effective governance structures. A Technical Working Group (referred to as the PLM Working Group) of key stakeholders from the MoH, its HPU and SNAP, and NERCHA was established by PLM from the onset of the project. This group provided regular opportunities for convening and sharing ideas, as well as reviewing ministry guidance related to AGYW's SRH that could further inform PLM's work.

You know what I like about the project, they are not telling us what to do. It's a matter of bringing our ideas together. They will look at us, say, ‘What are your views? How can we do it better? Because you are the ones who have been here, and you need to own the work here.’ (Public Sector Partner G)

The wide-step consultations, I think is key, because HIV is not a one-man show. There's been a lot of consultation here and there, and a lot of bringing minds together to say, ‘How best can we do these things.’ … Because we need to own up, as a sector ministry … (Public Sector Partner B)

The decision for PLM to focus on directly building the capacity of the Ministry's HPU was described as a conduit for local ownership and sustainability. While the HPU was viewed as having limited capacity, the opportunity for the department to have a novel project to build upon was appreciated.

The project [is] … a much-needed resuscitation of Health Promotion [Unit]. It's the one model that will make Health Promotion recognized as a health education department within the Ministry of Health. They haven't had many projects that directly feed into them … Finally, we have a project that is unique. A project that can then be transferred into the other programs within the Ministry of Health … (PLM Partner B)

People in various roles described how much they appreciated the opportunity to learn from Coca-Cola and their partners. The association with a global brand promoted buy-in and provided a useful metaphor for how the health sector would like their services to be valued.

Coca-Cola is a brand known world-wide. If they say they have a strategy, you sit up and you want to learn … it's telling me whatever message you have, keep telling the people. Don't tire like we have done in HIV. Our messaging should be targeted now. (Public Sector Partner A)

We’ve seen how Coca-Cola has stood the test of time … People are assured of the type of drink they’ll get, the taste they’ll get, and that it will be good … You’d want the target group when they see a certain sign in this facility, they’ll be assured of the package of services … (Public Sector Partner I)

### AGYW perspectives on an HIV prevention mindset

ZMET analyses resulted in a ‘Tree of Prevention’ framework summarising the major unconscious metaphors associated with HIV prevention illustrated by participants. One HIV negative participant compared her life to that of a strong, flowering tree:

… My life is like this tree. I will not contract AIDS because I depend on myself just like this tree depends on the roots. If water goes to the body, then the tree will live because the roots are the only source to get water … It does not depend on any other source to bring it water so if I also depend on myself and don't look for someone to buy me sweets, then I’m safe from contracting HIV. [The roots of the tree are] similar to me having a backbone … I cannot change my decision, I won't change my stance on contracting AIDS. Just like the tree, even if there is wind it only moves up here on the top but not at the bottom. It won't happen that I will be influenced by friends to change my mind. If I stand like this, then I will not be subjected to AIDS. (Urban young woman, 22 years old)

**Figure 2.**
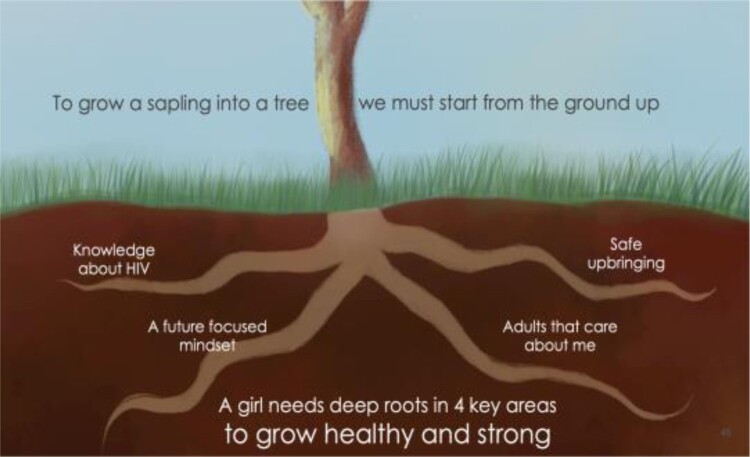
Four foundational factors contributing to HIV prevention derived from Olson Zaltman interviews with young women.

In this example, the tree ‘depends on its roots’ to provide a ‘backbone’ to withstand negative societal pressures and supply the tree, representing her sense of self, with water, symbolising economic and emotional resources. With these needs met, she does not have to rely on other means of survival. These include relationships with ‘blessers’, or older, married men who coerce young women into sexual activity in exchange for financial gain. Olson Zaltman described the roots of this tree as the four foundational factors for HIV prevention: access to *knowledge about HIV* and sexual health (metaphor A: darkness and light); a *safe upbringing* in early child and adolescent development, with access to *empathetic adults* including healthcare workers (metaphor B: connection), and support of a *future-focused mindset* (metaphor C: armoured plants like cacti and fierce animals) (Figure 2).

**Figure 3.**
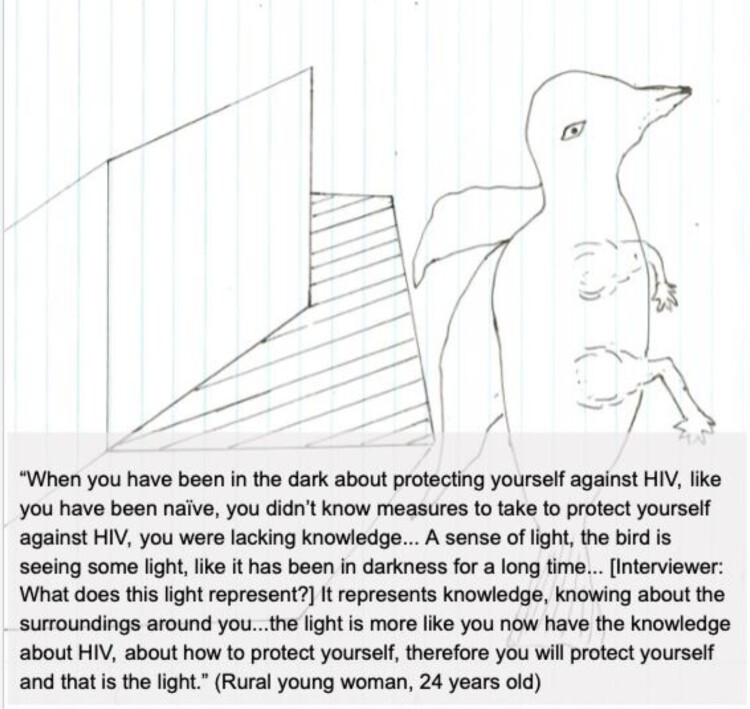
Participant drawing and quote illustrating Metaphor A.

Metaphor A: Darkness and Light. A lack of HIV prevention knowledge and education was compared to being trapped in darkness, as represented by one participant's drawing of a bird escaping a box (Figure 3).

AGYW displayed a solid understanding of the routes of HIV transmission, citing access to education and information resources via NGOs, schools, clinics, and churches as the source of this knowledge. The widespread availability of community-focused outreach was stressed, especially in semi-rural and rural communities. One 22-year-old young urban woman explained, ‘ … they [clinicians] talk to you and provide advice on how to stay healthy, protect yourselves … ’.

**Figure 4.**
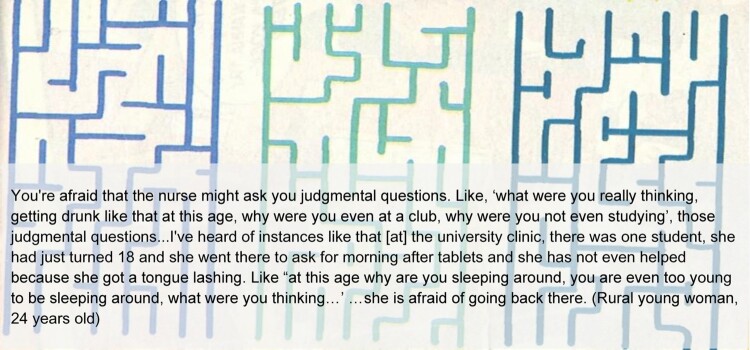
Participant provided image and quote illustrating Metaphor B.

Metaphor B: Connection. The ZMET analysis also highlighted the importance of access to a healthy upbringing and empathetic adults. Respondents illustrated these factors with images exemplifying connection or relationships, including families or parent–child couples pictured close together, implying loving bonds, emotional openness, and affection. Notably, these images often contained a sympathetic female figure. At the same time, AGYW were hesitant to seek the guidance of healthcare workers in schools and clinics due to the perception that some healthcare workers hold judgmental views of young women seeking SRH information or treatment, implying that young women should not be sexually active. These experiences emphasise the importance of human connection highlighted in the ZMET analysis, illustrating the need for open and empathetic communication with adults, including healthcare workers (Figure 4).

**Figure 5.**
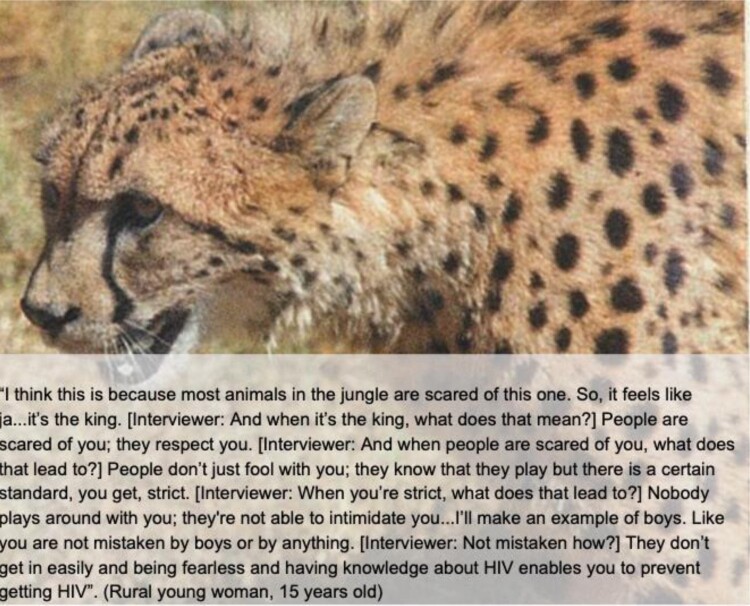
Participant provided image and quote illustrating Metaphor C.

Metaphor C: Armoured plants and fierce animals. In addition to knowledge and supportive adults, participants indicated that internalising and projecting strength was integral to defending oneself and avoiding the ‘wrong decisions’ that result in HIV infection. Participants represented themselves in the form of armoured plants and tenacious animals, such as lions and crocodiles, to withstand the pressures they faced. These representations indicated a need to resist forces that sway the participant away from HIV prevention, and highlighted a desire for control and agency (Figure 5).

These findings indicate that young women in eSwatini feel it is their responsibility to safeguard against the tensions that define gendered power dynamics, reflecting a future-focused mindset as essential to their protection. In addition, AGYW indicated the need for additional supports (education and supportive adults – light and connection) to reach their potential.

### Stakeholders described strong receptivity to market research findings

The market research results were well received by the PLM Technical Working Group, both for the robust insights they provided and the ease with which they could be translated into a creative marketing strategy. Stakeholders felt that the ZMET approach allowed young women to talk candidly about sensitive subjects, revealing the complex reality of young women's lives, and providing nuance that was not available from standard epidemiological surveys.

It [market research] kind of crystallizes the things that we kind of know because of the in-depth approach that was used. Painting pictures is more real to people than the facts. Like, we knew that girls were under pressure, and they fall victims … It was just one big problem that was hidden … It analyses how she feels, how she thinks, the pressures … it doesn't give you an all gloomy picture. It tells you all the other things—the aspirations, which are the things that we want to know because they are very helpful in terms of messaging. (Public Sector Partner F)

Stakeholders also valued the ease with which the market research findings were translated into a creative strategy. A respondent from the creative agency that spearheaded the communications strategy described the findings this way: ‘ … it [the market research] was almost gifted. It came up in a beautiful little box just people just had to unwrap it and run with it.’

Furthermore, the authenticity of the ZMET insights allowed the creative agency to craft an innovative marketing strategy that would resonate with young women in eSwatini:

 … We’ve got very close to these people without actually knowing them through the research … that for me helped a lot, and it helped us focus as well in terms of understanding, because it's something that we could refer back to in terms of its effectiveness from an execution perspective. I think it's helped a great deal from a creative and a strategic perspective. (Private Sector Partner B)

Stakeholders also described an appreciation for the up-front investment in understanding AGYW perspectives, prior to developing and implementing the strategy:

We rush to implementation. The strength of this [PLM approach] is that we take time to understand the audience, have clear techniques of how to reach those audiences, and its in-depth understanding and then develop things that are going to speak to the people as much as possible. (Public Sector Partner E)

### The evolution of the Girl Champ brand and COACH communication strategy

**Figure 6.**
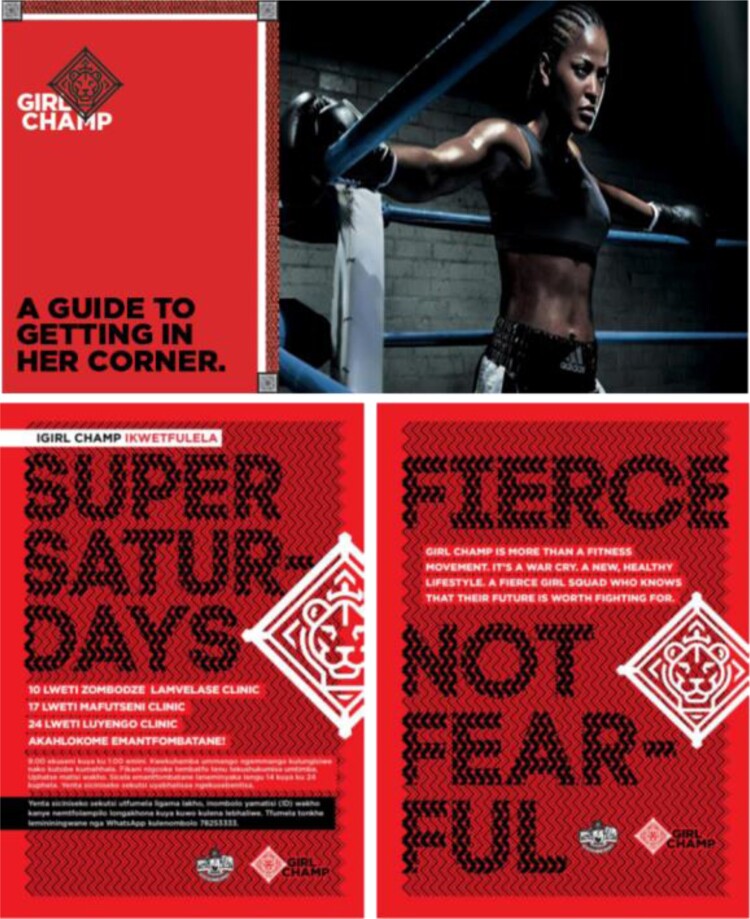
COACH guide (upper left) and Girl Champ promotional materials.

To leverage the aspirational identities of young women, and address their fears concerning judgmental health providers, it became apparent that a traditional mass media campaign would be insufficient to drive behaviour change. Olson Zaltman suggested tailoring health messaging and outreach to themes of personal empowerment within a realm of female-to-female advocacy, role modelling, and emotional support. FCB, in turn, developed a health and wellness brand that would empower young women to protect their health. The resulting Girl Champ brand is based on imagery of a lioness and a female boxer who can defend herself from life's pressures (Figure 6).

A Girl Champ can become informed and empowered to access services to protect her health. She joins other young women to form a powerful collective, in a girls-only safe space where stories are shared, support is given, and services are offered. During Girl Champ events known as ‘activations’ held at health facilities, AGYW are exposed to boxercise, yoga, and local entertainment; offered the opportunity to interact with nurses and frontline healthcare workers during candid Q&A sessions; and registered for health services. AGYW are offered the opportunity, but not forced, to access health consultations during the Girl Champ activations. Branded collateral (giveaways, pamphlets and posters advertising the events) evoke a lioness image, and incorporate local textiles to reinforce messages.

To enable consistent messaging with the Girl Champ brand and improve AGYW's experiences with healthcare, healthcare workers and staff at participating facilities are trained to become ‘COACHES’ a role and acronym to remind staff to be caring allies who provide health services and support in an objective way. The COACH acronym and curriculum was derived from the MoH's youth-friendly training curriculum for clinicians, and sought to orient healthcare providers into a role that is supportive yet maintains the professionalism required for youth-friendly care (Figure 6). The Girl Champ brand will be piloted in the Manzini region of eSwatini (results forthcoming).

## Discussion

There is an urgent need to close the gap in SRH outcomes among AGYW as compared to their male counterparts in Sub-Saharan Africa, and increasing emphasis on demand creation for HIV and SRH services through youth and community engagement. Furthermore, as eSwatini scales up pre-exposure prophylaxis (PrEP) and ‘test and start’ at the primary care level while also attempting to ‘build back better’ from COVID-19, there is both new opportunity and a critical need for strategic marketing that improves AGYW's demand for SRH services (Bärnighausen et al., 2020; Clark & Gruending, 2020; Walsh et al., 2020). Despite these needs, the potential to leverage private sector experience and expertise in strategic marketing to generate demand for services remains largely untested (Firestone et al., 2017). To characterise PLM's efforts in eSwatini, we brought together findings from two approaches: the Zaltman Metaphor Elicitation Technique (ZMET®) for market research with AGYW, and key informant interviews to characterise stakeholder experiences with private-sector engagement. Combining these approaches allows us to capture the novel insights into how to reach AGYW through application of a private sector strategic marketing process, and illuminates critical aspects of the partnership which facilitated translation of insights into action.

Although public-private partnerships are increasingly common in global health (Buse & Harmer, 2007; Curtis, Garbrah-Aidoo, & Scott, 2007; Kamugumya & Olivier, 2016; Linnander et al., 2018; Wong et al., 2015), there is considerable heterogeneity in both the public sector partners and the form of the partnerships (Allen & Bloomfield, 2016), and many focus on engagement of the private sector for service provision, rather than transfer of core business expertise (Linnander et al., 2017). Furthermore, few partnership approaches are adequately described in the peer reviewed literature. For example, in a systematic review, only 6 of 46 partnerships in LMIC settings had sufficient information on their activities and impact in the peer-reviewed and grey literature to allow for initial inclusion (Bhattacharyya et al., 2010). To our knowledge, our description of PLM's application of private sector strategic marketing processes to improve demand for adolescent SRH services is the first of its kind in the empirical literature. Over the project period, PLM achieved ownership and buy-in across diverse partners through the establishment and continuous engagement of a technical working group, commitment to develop capability within partnering government agencies, and the translation of proven marketing approaches from the private sector to public health priorities. These are key elements others have identified as necessary for successful public-private partnerships in the global health sphere (Buse & Harmer, 2007; Curtis et al., 2007; Kamugumya & Olivier, 2016; Maleka, 2017).

The ZMET research revealed a series of metaphors that, together, enable an HIV prevention mindset for AGYW: a tree with strong roots, the light of HIV prevention knowledge, connection with female caregivers, and the strength of armoured plants and fierce animals. These findings were valued by PLM stakeholders for providing relevant and nuanced insights and for the ease with which they were translated into a targeted marketing strategy. The resulting Girl Champ strategy included (1) a health, wellness, and empowerment brand for AGYW and (2) development of the COACH curriculum for healthcare workers to ensure facilities are equipped to provide consistent support and services. Notably, the Girl Champ brand exemplifies a gain-framing approach (Amico & Bekker, 2019) which leverages AGYW's future-oriented mindset and desire for light, connection, and strength. This is consistent with the growing literature suggesting that health messaging for adolescents, particularly concerning SRH and HIV prevention services, needs to be contextually situated, and emphasise individual wellness and social supports, rather than rely on fear-based, risk-oriented messaging (Eakle, Bourne, Jarrett, Stadler, & Larson, 2017; Hull, 2012; Mpondo et al., 2018; Ngidi, Moyo, Zulu, Adam, & Krishna, 2016; Sieving et al., 2017; Soon et al., 2013; Svanemyr, Amin, Robles, & Greene, 2015).

These results should be interpreted in light of several limitations. First, both study components are subject to social desirability bias (El Ansari & Weiss, 2005; Kelly, Soler-Hampejsek, Mensch, & Hewett, 2013). AGYW face heavy social stigma and taboos concerning pre-marital sex and HIV status and may fail to share complete or balanced descriptions of their experiences. The ZMET uses several approaches to mitigate this risk, including use of one-on-one interviews to avoid groupthink (Janis, 1971) or social dominance (Sidanius, 1999). ), and use of projective techniques such as personification and metaphor elicitations to promote deeper insights (Kopp, 1995; Weiser, 1993). Similarly, stakeholders interviewed may have had a vested interest in representing the partnership as a success. However, we identified common themes across stakeholder perspectives, sought rich descriptions of the phenomena being described, and probed for negative experiences and lessons learned (Lincoln & Guba, 1985). Second, the relatively small samples may limit the generalisability and deep attention to context which may limit transferability to other settings in sub-Saharan Africa. However, both research approaches revealed useful findings that resonate with other work on the development of effective public-private partnerships and engagement of clients in healthcare services (Buse & Harmer, 2007; Curtis et al., 2007). Third, we have not yet determined the impact of the resulting Girl Champ brand on demand for HIV/SRH services; the results of the pilot of the Girl Champ brand will be described in a future manuscript.

This paper brings together two complementary datasets to paint a robust picture of efforts to translate private sector strategic marketing principles to address public health issues. The associated stakeholder engagement fostered local receptivity to the market research approach, local ownership of the market research insights, and translation of market research findings into a brand strategy to improve AGYW's demand for SRH services. Findings may be useful for others seeking to adopt private-sector marketing approaches to improve AGYW's engagement with health services in other low- and middle-income settings.

## Supplementary Material

eSwatiniAppendices_SAHARAJ_021720PlnTxt_R1_TrackedChanges.docx
