# Supplementary material for: Project Last Mile and the development of the Girl Champ brand in eSwatini: engaging the private sector to promote uptake of health services among adolescent girls and young women
Source: SAHARA J. 2021 Mar 8;18(1):52–63. doi: 10.1080/17290376.2021.1894224 (PMC7946024; doi:10.1080/17290376.2021.1894224)
Supplement: eSwatiniAppendices_SAHARAJ_021720PlnTxt_R1_TrackedChanges.docx [file RSAH_A_1894224_SM7722.docx]

Supplemental File

**Methods**

ZMET is based on several fundamental principles about the human mind and how people make decisions. The first principle is that most thought occurs below conscious awareness (Damasio, 1994; Kahneman, 2011; Nørretranders, 1998). Therefore, asking people direct questions will likely lead to them providing shallow rationalizations for their behavior. Second, humans think in images or neural patterns, not words (Damasio, 1994, 1999; Pinker, 1995, 1997), and thus visual and other sensory imagery can help researchers better understand the inherently imprecise and typically nonliteral terms that people use to describe their thoughts and feelings. Finally, metaphor is vital to creating and understanding meaning. As defined by George Lakoff (1993), metaphor is the conceptualization of one mental domain in terms of another and is a key lens through which we understand and interpret our world (Gibbs, 2008; Kövecses, 2005; George. Lakoff, 1980). ZMET leverages the power of metaphor and other nonliteral expressions to uncover people’s unconscious emotional framings and motivations (Zaltman, 1997, 2003; Zaltman & Coulter, 1995; Zaltman & Zaltman, 2008).

**Final codebook for stakeholder data analysis**

| **Code Name** | **Definition** |
| --- | --- |
| **100. Context** |  |
| 101. Context | Enabling/disabling regulatory, legal and political factors; policy landscape; country context and culture; politics |
| 102. Context – Gender/Social Norms | Refers to the social and cultural context as it pertains to gender equity, societal norms, vulnerability, cultural mores, traditions, power imbalance [that may influence demand creation but not necessarily] |
| **200. PLM Process** |  |
| 201. Knowledge transfer | Between Coke/PLM and other partners, sharing expertise, capacity building; actual transfer of knowledge or capabilities. Excludes private sector inputs |
| 202. Private sector inputs_Technical | Technical expertise that PLM/Coke is providing to public sector partners, such as tools, data, analytic strategies, soft inputs, etc. Excludes marketing and communication inputs |
| 202.a. Private sector inputs_Marketing&Comm | Marketing and communication inputs being provided by PLM/Coke to public sector partners. Includes assistance with branding, franchising, market research, creative design, demand creation, etc. |
| 202.b. Private sector inputs_Expectations | Expectations about what the private sector/Coke will bring in terms of technical inputs, training, innovations, marketing, capacity building, tools, software, etc. that may or may not be met. |
| 203. Adaptation/flexibility | Adaptations/shifts in response to circumstances, examples of how something was adapted to fit circumstances, context |
| 204. Networks | Boundary spanners, active use of pre-existing relationships to make connections, development and dynamics of relationships that contribute to the work |
| 205. Assessing and aligning “fit” between PLM and external partners | Assessment of the landscape, and planning for how PLM is/will be involved; Coordinating and finding complementary roles, complementary expertise; addressing role conflict/overlap and competing demands/priorities amongst partners; relationship-building and stakeholder engagement. Can also include aligning with donor priorities. |
| 206. Process | Description of processes through which PLM contributes, the tasks and work done by PLM. The steps PLM has followed to reach their current involvement. Might include knowledge transfer, but not necessarily. |
| 207. Data sources and evaluations | Any metrics or data that has been used in evaluating different components of PLM |
| 208. Historical explanations | Explanations for how partnerships, processes, other aspects came to be. |
| 209. Impact/sustainability | References to long-term results or impact of PLM project, including sustainability of the project after PLM implementation. Could include discussion of factors that may influence long-term results and sustainability. |
| 210. Boundary spanner | Refers to a particular person in the PLM partnership who is able to communicate across public and private sectors |
| 211. Governance | Refers to PLM project oversight, roles and responsibilities, planning and deadlines; also can refer to how PLM is governed by external structures and situated within organization |
| 212. Route Optimization | Refers to PLM workstreams related to optimizing routes for distribution, including geomapping, supply chain logistics, related software and tools |
| 213. Capacity Building | Refers to PLM workstreams related to specific capacity building, such developing organization structures, job descriptions, and performance management processes for the public sector |
| 214. Outsourced Distribution | Refers to PLM workstreams related to facilitating and negotiating outsourced distribution |
| 215. Demand Creation | Refers to PLM workstreams related to facilitating demand creation, including market segmentation and research, creative agency development, strategic communications, organization development, messaging |
| 215a. Demand Creation – Research | Refers to the process of research to inform messaging and communication strategy, selection of the agency, can refer to quantitative or qualitative data collection and related procedures, research findings, receptivity to research findings |
| 215b. Demand Creation – Communication Strategy | Refers to the process of creating and developing the communication strategy, selection of the agency, can refer to how the research findings informed the communication strategy, how the communication strategy will be rolled out (media placement, etc.), the message and media for the communication strategy, receptivity to the proposed strategy |
| 215c. Demand Creation – Implementation | Refers to the process of putting the communication strategy into practice at the health facilities, how it will be operationalized, the sell-in for the health facilities, how it will be integrated into existing initiatives with existing personnel, training and capacity building, site selection |
| 216. Innovation | Refers to innovation or lack thereof; when a respondent refers to something as a new way of doing things or explicit reference to an innovation that was contributed by PLM. Can also refer to expectations for innovation and whether the partnership met that expectation (and how). |
| **300. Influences on PLM process** |  |
| 301. Inter-sectoral communication | Communication across organizations/partners, such as between ministries of health and private sector partners. Excluded: communication within a single unit (department, level of system, or organization). |
| 302. Communication within units | Communication within a department, level of the system, or organization. |
| 303. Organizational culture | Descriptions of learning and problem-solving, ways of working together, commitment to organization, team work. |
| 304. Buy-in and ownership | Extent to which individuals at different levels of the system feel ownership and want to support or be a part of PLM or PLM-supported programs, part of team. |
| 305. Trust ~~and motivation~~ for partnerships | Feelings of trust (or lack thereof) between partners, in the development of relationships and partnership. Also can indicate alignment on good will, public benefit or lack thereof (i.e., potential for conflict of interest). |
| 306. Momentum | References to the pace at which PLM’s work has moved; ebbs and flows in progress. |
| 307. Funding/Resources | Refers to availability of financial/monetary resources as well as human resources. |
| 308. External communications and advocacy | Refers to communication about and dissemination of PLM’s work to organizations and stakeholders outside of the partnership |
| 309. Bureaucracy and Contractual Considerations | Refers to contractual issues, bureaucratic processes, ‘red tape’, regulatory requirements and how they affect the partnership |
| **900. Overarching codes** |  |
| 901. Change | Any changes as a result of PLM’s involvement; Changes to CCMDD; To be applied in addition to whatever changed (communication, organizational culture, etc.) |
| 902. Barriers | Anything that has inhibited PLM, partnerships, CCMDD. To be applied in addition to whatever the barrier was (communication, organizational culture, etc.) |
| 903. Facilitators | Anything that has facilitated PLM, partnerships, CCMDD, helped in overcoming challenges. To be applied in addition to whatever the facilitator was (communication, organizational culture, etc.) |
| 904. Great quote | Quotations that are particularly illustrative of particular themes. |
| 905. Successes | Anything described as a successful outcome of PLM. May or may not also be coded as change. |
| 906. Feedback and Recommendations | Information that might be useful to provide as feedback to partners (delivery team, PMO, steering/working committees). Could be double-coded with a code that is specific to the recommendation, but not necessary. |

**For references, see main manuscript references**
